# Intraoperatively Diagnosed Tracheal Tear after Using an NIM EMG ETT with Previously Undiagnosed Tracheomalacia

**DOI:** 10.1155/2013/568373

**Published:** 2013-03-27

**Authors:** Minal Joshi, Simon Mardakh, Joel Yarmush, H. Kamath, Joseph Schianodicola, Ernesto Mendoza

**Affiliations:** ^1^Department of Anesthesiology, New York Methodist Hospital, 506 6th street, Brooklyn, NY 11215, USA; ^2^Department of Surgery, New York Methodist Hospital, 506 6th Street, Brooklyn, NY 11215, USA

## Abstract

Tracheal rupture is a rare complication of endotracheal intubation. We present a case of tracheal rupture that was diagnosed intraoperatively after the use of an NIM EMG endotracheal tube. A 66-year-old female with a recurrent multinodular goiter was scheduled for total thyroidectomy. Induction of anesthesia was uncomplicated. Intubation was atraumatic using a 6 mm NIM EMG endotracheal tube (ETT). Approximately 90 minutes into the surgery, a tracheal tear was suspected. After confirming the diagnosis, conservative treatment with antibiotic coverage was favored. The patient made a full recovery with no complications. Diagnosis of the tracheal tear was made intraoperatively, prompting early management.

## 1. Introduction

Tracheal rupture is a rare iatrogenic complication, most commonly due to blunt trauma outside the hospital setting. It is occasionally a complication of surgical manipulation of the trachea. It can also complicate orotracheal intubation due to the tip of endotracheal tube (ETT) getting caught in the fold of posterior trachea during insertion. Diagnosis usually waits until after extubation based on clinical suspicion and confirmed by bronchoscopy. If not properly managed, severe respiratory distress and even death may result. We present a case of tracheal rupture that was diagnosed intraoperatively via bronchoscopy and managed conservatively.

## 2. Case Presentation

**Figure 1:**
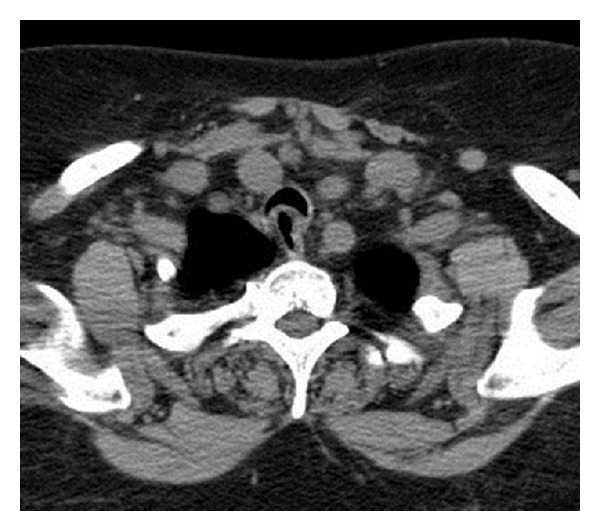
Preoperative CT scan showing deviation and compression of trachea.

A 66-year-old female with a history of a subtotal thyroidectomy 20 years ago was scheduled for a total thyroidectomy due to a recurrent symptomatic multinodular goiter. She presented with increasing dysphagia, dyspnea, and a nontender neck mass. Preoperative fine-needle aspiration showed benign follicular hyperplasia. CT scan revealed an enlarged thyroid nodule on the left lobe measuring 4.7 × 3.1 cm with deviation of the trachea to the right (Figure 1). Other significant medical history included diabetes, hypertension, and hyperlipidemia.

After induction of anesthesia with propofol and succinylcholine, a 6 mm Medtronic nerve integrity monitor (NIM) EMG ETT was inserted over a stylet for an uneventful intubation. 

Approximately 90 minutes into the surgery, a gurgling noise was perceived from the operative site. This was followed by an increase in inspiratory peak pressure and desaturation to 85%. Tube placement was confirmed by laryngoscopy, and the cuff was further inflated with 2 mL of air to minimize leaks. Fiber optic bronchoscopy revealed blood around the ETT, which was suctioned resulting in improved respiratory parameters.

**Figure 2:**
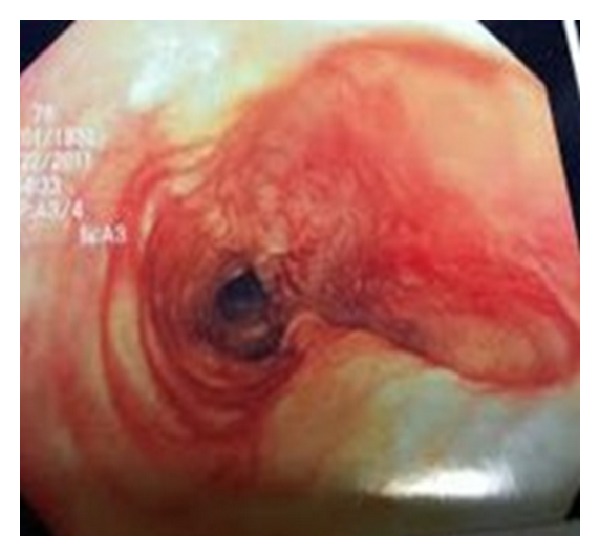
Tracheal tear on posterior wall on intraoperative flexible bronchoscopy.

After removal of the thyroid gland, the anterolateral aspect of the trachea was examined and palpated. No anterolateral tear was found. A repeat flexible bronchoscopy revealed a 5 cm tracheal tear on the posterior wall approximately 2 cm above the carina (Figure 2). Tracheomalacia was noted distal to the ETT. Flexible endoscopy performed at this time to rule out esophageal tear was unrevealing. The surgical incision was closed 4 hours after the start of the procedure. The NIM EMG ETT was exchanged for a regular ETT. The patient was then transferred to the ICU.

**Figure 3:**
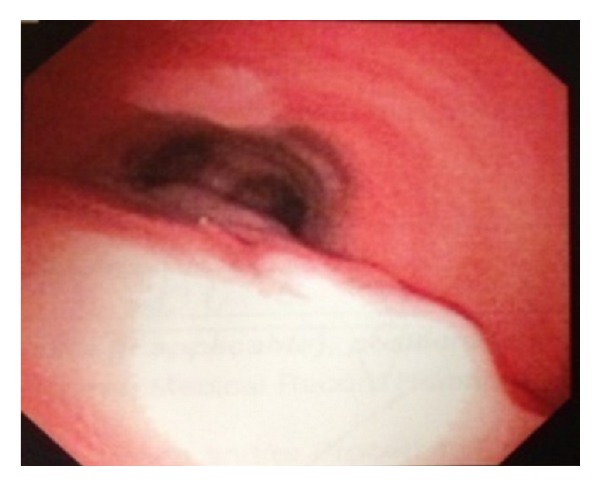
Healing with evidence of granulation tissue on POD 8.

**Figure 4:**
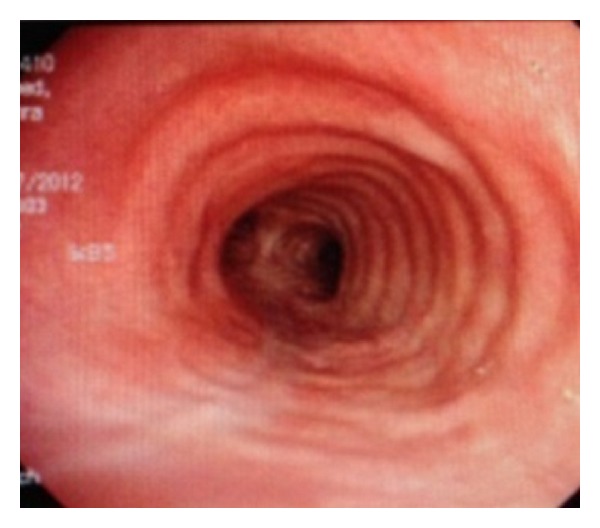
Bronchoscopy showing tracheomalacia on postoperative week no. 17. (note: complete healing).

On CMV (PIP of 25 cm H_2_O, FiO_2_ 100%), the ABG showed pH 7.32, pCO_2_ 40, pO_2_ 96, and O_2_ saturation 96%. A chest X-ray revealed no evidence of pneumomediastinum or subcutaneous emphysema. In the SICU, she was started on IV antibiotics and was monitored for respiratory distress. On postoperative day (POD) 1, she was put on CPAP trials and a flexible bronchoscopy confirmed the intraoperative findings. Subsequently, she was extubated and placed on supplemental oxygen via face mask. A chest X-ray was unchanged other than a new mild pleural effusion. A flexible bronchoscopy done on POD 5 showed the tracheal rupture in the initial healing phase. She was transferred to the general floor on POD 6. A flexible bronchoscopy done on POD 8 showed further healing of the tracheal rupture (Figure 3). The remainder of her hospital course was uneventful, and she was discharged on POD 12. Followup as an outpatient (week 17) revealed a fully healed tracheal tear with evidence of tracheomalacia (Figure 4).

## 3. Discussion

One of the rare complications of intubations is a tracheal tear. Estimates of its incidence in the last decade have ranged from approximately 0.05% to 0.37% of all orotracheal intubations [1]. A variety of risk factors both mechanical and anatomical predispose a patient to such an injury. Notable risk factors include a difficult intubation, the use of a stylet, prolonged intubation, and over inflation of the cuff. Patient risk factors include advanced age, female sex, tracheal anomalies, history of chronic obstructive pulmonary disease, and congenital tracheal anomalies such as tracheomalacia [2–4].

It presents more frequently in women owing to a weaker pars membranosa and the use of endotracheal tubes of a larger size than appropriate for women [5]. Usually diagnosed postoperatively, common symptoms include subcutaneous emphysema, mediastinal emphysema [6], pneumothorax, and hemoptysis. Tears under 2 cm are often asymptomatic [3]. Chest X-ray and CT scan show pneumomediastinum and/or pneumopericardium [3, 6]. Ventilation studies can show a persistent air leak. Bronchoscopy is needed for definitive diagnosis and to evaluate the extent of the tear.

Current management options for tracheal rupture are divided into conservative and surgical therapy. In most iatrogenic cases, the wound is limited, and the airway is maintained, allowing for conservative management. In traumatic injuries with loss of continuity in the proximal and distal part of the trachea, surgical intervention is required [3]. Surgical intervention is also recommended for complicated cases with rupture that extends into the main bronchi [4]. A study done by Cuesta et al. showed that conservative management is effective regardless of causation, size, or site of injury [7]. Conservative therapy includes orotracheal intubation, IV antibiotics, and parenteral nutrition. The cuff pressure of the ETT must be kept low and the tidal volume 5-6 mL/kg to expedite healing of the tear [6]. Extubation must be performed as soon as possible. Bronchoscopy should be utilized to monitor healing of the tear.

In this patient, the tracheal wall may have been compromised because of her undiagnosed tracheomalacia. The tracheomalacia, in turn, may have been a sequela of the original surgery and her long-standing goiter. Although in this case intubation was atraumatic, the use of an NIM EMG ETT in conjunction with undiagnosed tracheomalacia may have precipitated the tear. The NIM EMG tube uses a silicone elastomer cuff to allow better tracheal conformity and minimal tissue trauma. The designed elasticity may predispose the silicone-based cuff to asymmetrically inflate at very high cuff pressure [8].

Diagnosis of tracheobronchial injury can be made in a wide range of time periods. However, a delay in the diagnosis has been shown to have poor outcomes [4]. In our patient, the diagnosis of the tear was made intraoperatively, allowing a focused early management.
